# Continuous Positive Airway Pressure Treatment and Hypertensive Adverse Outcomes in Pregnancy: A Systematic Review and Meta-Analysis

**DOI:** 10.1001/jamanetworkopen.2024.27557

**Published:** 2024-08-13

**Authors:** Yi-Chieh Lee, Yun-Chen Chang, Liang-Wei Tseng, Wan-Ni Lin, Chun-Ting Lu, Li-Ang Lee, Tuan-Jen Fang, Wen-Nuan Cheng, Hsueh-Yu Li

**Affiliations:** 1 Department of Otolaryngology–Head and Neck Surgery, New Taipei Municipal Tucheng Hospital (Built and Operated by Chang Gung Medical Foundation), New Taipei City, Taiwan; 2 London School of Hygiene and Tropical Medicine, London, United Kingdom; 3 Sleep Center, Department of Otolaryngology–Head and Neck Surgery, Chang Gung Memorial Hospital at Linkou, Taoyuan, Taiwan; 4 Division of Chinese Acupuncture and Traumatology, Center of Traditional Chinese Medicine, Chang Gung Memorial Hospital, Taoyuan, Taiwan; 5 College of Medicine, Chang Gung University, Taoyuan, Taiwan; 6 Department of Sports Sciences, University of Taipei, Taipei, Taiwan

## Abstract

**Importance:**

Pregnancy may contribute to the development or exacerbation of obstructive sleep apnea (OSA) and increase the risk of gestational complications. Continuous positive airway pressure (CPAP) is the first-line and criterion standard treatment for OSA and is regarded as the most feasible choice during pregnancy. However, the association between CPAP therapy in pregnant women with OSA and reduced gestational complications remains inconclusive.

**Objective:**

To investigate the association between CPAP therapy in pregnant women with OSA and the reduction of adverse hypertensive outcomes during gestation.

**Data Sources:**

Keyword searches of PubMed, Embase, and the Cochrane Database of Systematic Reviews and Clinical Trials were conducted from inception to November 5, 2023.

**Study Selection:**

Original studies reporting the treatment effect of CPAP use on lowering hypertension and preeclampsia risks in pregnant women with OSA were selected.

**Data Extraction and Synthesis:**

The Preferred Reporting Items for Systematic Reviews and Meta-Analyses (PRISMA) guideline was followed in the reporting of reviews. Data were independently extracted by 2 authors. Random-effects model meta-analyses were performed and risk ratios (RRs) reported. Subgroup analysis, meta-regression based on age and body mass index (BMI; calculated as weight in kilograms divided by height in meters squared), and publication bias assessment were also conducted.

**Main Outcome and Measures:**

The primary outcome was the RR of gestational hypertension and preeclampsia between pregnant women with OSA receiving CPAP treatment and those who did not receive CPAP treatment.

**Results:**

Six original studies in 809 participants (mean age, 31.4 years; mean BMI, 34.0) were identified and systematically reviewed for meta-analysis. The pooled results showed significant differences between the intervention (CPAP use) and the control (non-CPAP use) groups in reducing the risk of gestational hypertension (RR, 0.65; 95% CI, 0.47-0.89; *P* = .008) and preeclampsia (RR, 0.70; 95% CI, 0.50-0.98; *P* = .04). Meta-regression revealed that patients’ age (coefficient, −0.0190; *P* = .83) and BMI (coefficient, −0.0042; *P* = .87) were not correlated with reduction of risk of hypertension and preeclampsia.

**Conclusions and Relevance:**

These findings suggest that implementing CPAP treatment in pregnant women with OSA may reduce the risk of gestational hypertension and preeclampsia.

## Key Points

**Question:**

Is there an association between continuous positive airway pressure (CPAP) and lowered risk of gestational hypertension and preeclampsia for pregnant women with obstructive sleep apnea (OSA)?

**Findings:**

In this systematic review and meta-analysis of 6 studies including 809 pregnant women with OSA, the pooled results showed a significant reduction in the risk of gestational hypertension (35%) and preeclampsia (30%).

**Meaning:**

These findings suggest that implementing CPAP treatment in pregnant women with OSA may reduce the risk of adverse gestational hypertensive outcomes.

## Teaser

This systematic review and meta-analysis investigates the association between continuous positive airway pressure (CPAP) therapy and reduction of adverse hypertensive outcomes in pregnant women.

## Introduction

Obstructive sleep apnea (OSA) is a sleep disorder characterized by repetitive airway obstruction during sleep. It is estimated to affect 10% to 30% of the middle-aged adult (30-69 years) population worldwide and is on the rise due to the global increase in obesity.^1,2^ If left untreated, OSA is known to be associated with several critical health consequences, including hypertension, coronary heart disease, diabetes, and stroke.^3,4,5,6,7,8^

Pregnant women can develop or experience worsening of OSA, which may be attributable to 4 major factors: hormonal changes, anatomic changes, weight gain, and fluid retention.^9,10^ During pregnancy, an elevated estrogen level can cause hyperemia of the upper airway and nasal mucosa, which may decrease airway size and pharyngeal dimensions. Studies have shown a reduction in airway size in pregnant women compared with the general population.^10,11^ Additionally, weight gain and fluid retention during pregnancy may further contribute to narrowing of the pharyngeal airway and nocturnal airway obstruction.^12^ Notably, frequent snoring, a hallmark symptom of OSA, is reported by 15% to 25% of pregnant individuals, with up to one-third experiencing snoring in the third trimester.^13^ Among pregnant women, the prevalence of sleep-disordered breathing increases as the pregnancy progresses, rising from less than 5% in early pregnancy to approximately 10% in mid-pregnancy.^13^ Elevated rates are particularly notable in individuals with additional risk factors for OSA, such as obesity, advanced maternal age, hypertension, or a history of preeclampsia.^14^

Large cohort studies have shown that pregnant women with OSA face a substantially higher risk of pregnancy-specific complications, such as gestational hypertensive syndromes, preeclampsia, and gestational diabetes, compared with those without.^9,15,16^ Two major contributing factors are intermittent hypoxia and fragmented sleep. These conditions can affect both the mother and the developing fetus. Intermittent drops in oxygen levels can lead to oxidative stress, inflammation, and endothelial dysfunction, all of which lead to an imbalance of proangiogenic and antiangiogenic factors and are considered the underlying mechanism of preeclampsia.^12^ In addition, the disrupted sleep patterns associated with OSA may contribute to increased sympathetic activity and hormonal and metabolic changes, potentially leading to gestational hypertension. Gestational hypertension syndromes and preeclampsia can be life-threatening if left untreated, with the possibility of more severe complications, such as eclampsia. Moreover, mothers with OSA have a higher incidence of congenital anomalies in their babies, with a prevalence exceeding 17%, which is significantly higher than for mothers without OSA.^17^

For OSA treatment, the main options include lifestyle modification, exercise, continuous positive airway pressure (CPAP), mandibular advancement devices, and surgery.^1^ Among these treatment options, CPAP is considered the first-line and criterion standard therapy and regarded as the most feasible choice before, during, and after pregnancy due to the characteristic of noninvasiveness.^18,19,20^

Studies have shown a substantial reduction in the incidence of hypertensive syndromes in pregnant women with OSA after using CPAP during pregnancy.^21,22^ However, some recent randomized clinical trials (RCTs) did not demonstrate a positive effect of CPAP treatment.^23^ Currently, there is still no consensus guideline on the management of OSA during pregnancy due to underestimated consequences and vulnerability of the population. As a result, the effectiveness of CPAP treatment in pregnant women with OSA remains inconclusive. Our study aimed to review all available RCTs and observational studies (non-RCTs) with data to provide an updated meta-analysis on the association between CPAP treatment and reduction of adverse maternal outcomes in pregnant women with OSA. Additionally, we discuss the indication for implementing CPAP treatment in pregnant women with OSA and the optimal timing for initiating CPAP treatment during pregnancy.

## Methods

### Data Sources and Study Selection

This systematic review and meta-analysis adhered to the Preferred Reporting Items for Systematic Reviews and Meta-Analyses (PRISMA) guidelines, and the predefined study protocol has been published on the International Platform of Registered Systematic Review and Meta-Analysis Protocols.^24^

We conducted a systematic search through PubMed, Embase, and the Cochrane Database of Systematic Reviews and Clinical Trials without language restrictions (all searched from inception to November 5, 2023). Briefly, the following search terms were used: (OSA or OSAS or OSAHS or SDB or SRBD or OSDB or SAHS) OR “snoring” OR “sleep disordered breathing” OR “sleep apnea”) AND (“gestation*” OR “gestational hypertension” OR “pre-eclampsia” OR “eclampsia”) AND (“pregnancy“). References of relevant articles were also searched for potentially eligible studies. After removing duplicates, titles and abstracts were independently reviewed by ^2^ authors (Y.-C.L. and Y.-C.C.) to select eligible studies for full-text review. Discrepancy was resolved by discussion between the 2 authors to achieve consensus.

### Inclusion and Exclusion Criteria and Data Extraction

Inclusion criteria were as follows: (1) pregnant women with OSA confirmed by polysomnography or home sleep test, (2) treatment with CPAP, (3) clearly defined experimental and control groups, and (4) outcome measurements that included risk of composite hypertensive outcomes (eg, gestational hypertension or preeclampsia). Exclusion criteria were as follows: (1) the study did not contain the risk of hypertensive outcomes; (2) patients received treatment other than CPAP; and (3) the study was classified as an article review, protocol, letter, poster, conference summary, case report, or editorial. Two authors (Y.-C.L. and Y.-C.C.) independently extracted information from each publication, including first author, publication year and type, study design, country where the study was performed, number of patients, age, body mass index (BMI [calculated as weight in kilograms divided by height in meters squared]), treatment duration, randomization process, and outcome measurement.

### Risk-of-Bias and Quality Assessment

The risk-of-bias and quality assessment were conducted by Y.-C.L. and Y.-C.C. using the Cochrane risk-of-bias tool, version 2^25^ for RCTs and the Newcastle-Ottawa Scale^26^ for observational studies. The Cochrane risk-of-bias tool includes 3 quality levels: high, some concerns, and low. Specifically, in the intervention adherence section, there are 2 options for literature assessment: intention to treat (reflecting intervention assignment) and per protocol (representing intervention adherence). For this meta-analysis, we opted for the per-protocol evaluation as it aligns best with the design of our included studies. The assessment incorporated 5 domains: the randomization process, deviations from the intended interventions, missing outcome data, outcome measurement, and selection of the reported results. In the event of a disagreement, the corresponding author (H.-Y.L.) was consulted to achieve consensus.

The Newcastle-Ottawa Scale rates studies from 0 to 9 points across 3 domains. Maximum points of 4, 3, and 2 are allocated for the population selection, outcome assessment, and comparability domains, respectively. Studies scoring a total of at least 7 points were considered to be of high quality.

### Statistical Analysis

All statistical analyses and plotting were conducted using RStudio, version 2022.07.2 (R Foundation) with packages meta and metabin. The random-effects model was used because the outcomes could vary between studies. Since the outcome measurements were incidence risk, the risk ratios (RRs) and 95% CIs were used in forest plots. A 2-sided *P* < .05 was considered statistically significant.

To measure heterogeneity among the studies, the Cochran *Q* test and *I*^2^ statistic were used. A *Q* test *P* < .05 or *I*^2^ > 50% indicated the presence of heterogeneity. Subgroup analysis was also conducted to evaluate heterogeneity. Sensitivity analysis was performed by omitting each study to evaluate the stability of results. To assess the risk of publication bias, the trim-and-fill method was used because fewer than 10 studies were included in the analysis.

## Results

### Study Characteristics

**Figure 1.**
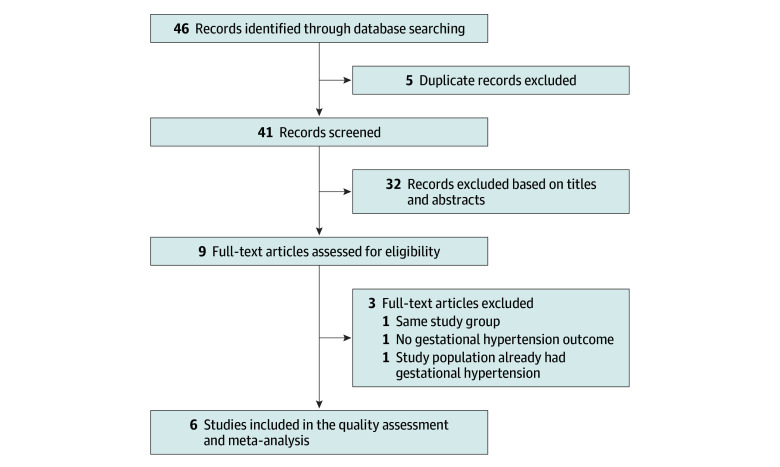
PRISMA Flow Diagram for the Meta-Analysis

A flow diagram of the research screening process is presented in Figure 1. A total of 46 articles were retrieved through the database search. After removing duplicate studies, the titles and abstracts of the remaining 41 articles were screened, and 32 were excluded. After reviewing the full texts of the remaining 9 articles, 3 were excluded as not meeting our study’s inclusion criteria, leaving 6 for the meta-analysis.^21,22,23,27,28,29^

The characteristics of the included studies are summarized in the Table. All were published since 2018 and were conducted in the US,^21,28,29^ Thailand,^27^ Japan,^23^ and Serbia.^22^ In total, 809 participants were included (mean [SD] age, 31.4 years; mean [SD] BMI, 34.0). The studies include RCTs,^23,27,28,29^ a retrospective study,^21^ and a prospective cohort study.^22^

**Table.** Summary of the Included Trials

Source	Study design	Location	No. of participants	Timing of CPAP use	Duration of treatment	Compliance	Control group treatment	Measurement criteria		Key findings
								Gestational hypertension	Preeclampsia	
Facco et al,^29^ 2023	RCT, no masking	US	85	Not mentioned	Not mentioned	Days >4 h use: phase 1, 4%; phase 2, 18%	Sham CPAP or sleep hygiene	Not mentioned	Not mentioned	Autotitrating CPAP in pregnancy did not reveal any differences in a composite primary cardiometabolic risk profile between the treatment groups
Tantrakul et al,^27^ 2023	RCT, no masking	Thailand	311	14-16 wk	Until delivery	32.7%	Standard care	ACOG guideline	ACOG guideline	CPAP decreased hypertension and preeclampsia in patients with mild to moderate OSA
Kalkhoff et al,^23^ 2022	RCT, no masking	Japan	187	6-16 wk and 27-33 wk	Not mentioned	2% (1 of 64 d) to 43% (6 of 14 d)	Standard care	Hypertension diagnosed after 20 wk	Gestational hypertension with proteinuria	There was no significant difference in hypertension and preeclampsia
Rice et al,^21^ 2023	Retrospective cohort	US	100	Mean (SD), 23.4 (7.4) wk	Not mentioned	Mean (SD), 84% (15%) (days >4 h use)	Standard care	ACOG guideline	ACOG guideline	CPAP therapy reduced the risk of hypertension among pregnancies affected by OSA
Stajić et al,^22^ 2022	Prospective cohort	Serbia	91	24-28 wk	4 wk	Mean (SD), 6.1 (1.0) h per night	Standard care	Hypertension diagnosed after 20 wk	Gestational hypertension with ≥1 new-onset conditions^a^	Treatment with CPAP significantly reduced the incidence of severe forms of hypertension and maternal-fetal outcomes in pregnant women with OSA
Chirakalwasan et al,^28^ 2018	RCT, no masking	US	36	24-34 wk	2 wk	46.7%	Standard care	NA	Not mentioned	CPAP for 2 wk in women with GDM and OSA did not result in improved glucose levels, but insulin secretion improved in those adherent to CPAP

Abbreviations: ACOG, American College of Obstetricians and Gynecologists; CPAP, continuous positive airway pressure; GDM, gestational diabetes mellitus; NA, not applicable; OSA, obstructive sleep apnea; RCT, randomized clinical trial.

^a^ Significant proteinuria (>0.3 g/24 hours or albumin:creatinine ratio ≥30 mg/mmol), thrombocytopenia (platelet count <100 000/μL), impaired liver function (elevated blood levels of liver transaminases to twice the normal concentration, with or without right-upper-quadrant abdominal pain), the new development of kidney insufficiency (elevated serum creatinine >1.1 mg/dL or a doubling of serum creatinine in the absence of other kidney diseases), pulmonary edema, or new-onset neurologic complication (eclampsia, altered mental status, blindness, stroke, clonus, severe headaches).

In all 6 studies, events for hypertensive disorder, including gestational hypertension and preeclampsia, were reported. The intervention groups were treated with CPAP, and the control groups were treated using standard care for pregnant women. In the study conducted by Rice et al^21^ and Stajić et al,^22^ participants were divided into 3 groups: without OSA, with OSA and CPAP compliant, and with OSA and CPAP not compliant or conservative treatment. For our meta-analysis, we selected OSA and CPAP compliant as the intervention group and OSA and CPAP not compliant or conservative treatment as the control group. This selection was made to enhance our assessment of the efficacy of CPAP in pregnant women with OSA. Adherence to CPAP treatment ranged from less than 10% (poor adherence)^23^ to 85% adherence.^21^

### Quality Assessments

The quality assessment of the RCTs using the Cochrane risk-of-bias tool is detailed in the eTable in Supplement 1. All 4 RCTs were homogeneously designed and open-label studies.^23,27,28,29^ However, the assessment was not influenced by knowledge intervention. All the RCTs were labeled as low risk.

Newcastle-Ottawa Scale scores for non-RCT study bias assessment are presented in the eTable in Supplement 1. The included studies^21,22^ met most of the quality assessment criteria except for comparability, and both studies had a total score of 6 points.

### Primary Outcomes

**Figure 2.**
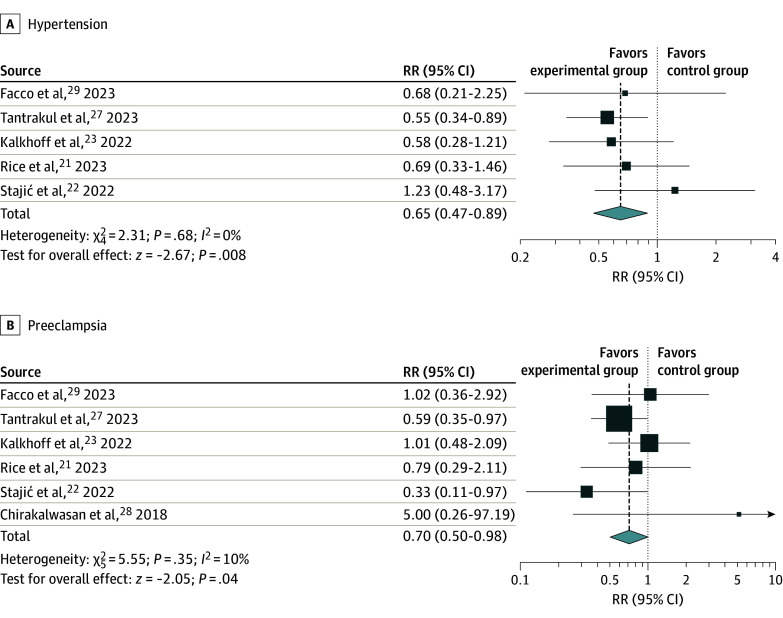
Pooled Risk Ratios (RRs) for Continuous Positive Airway Pressure Treatment and Hypertensive Adverse Outcomes in Pregnant Women The 95% CI was calculated using random-effects model meta-analysis.

To evaluate the use of CPAP in pregnant women with OSA, the events of gestational hypertension and preeclampsia in the intervention and control groups were used as the primary outcomes. Events of hypertension in 5 studies^21,22,23,27,29^ and events of preeclampsia in all 6 studies were pooled for meta-analysis. The results show that the use of CPAP was associated with a significantly reduced risk of developing gestational hypertension (RR, 0.65; 95% CI, 0.47-0.89, *P* = .008; *I^2^* = 0%) (Figure 2A). Additionally, CPAP use was associated with a significant decrease in the risk of developing preeclampsia (RR, 0.70; 95% CI, 0.50-0.98, *P* = .04; *I^2^* = 10%) (Figure 2B). Low heterogeneity was observed.

**Figure 3.**
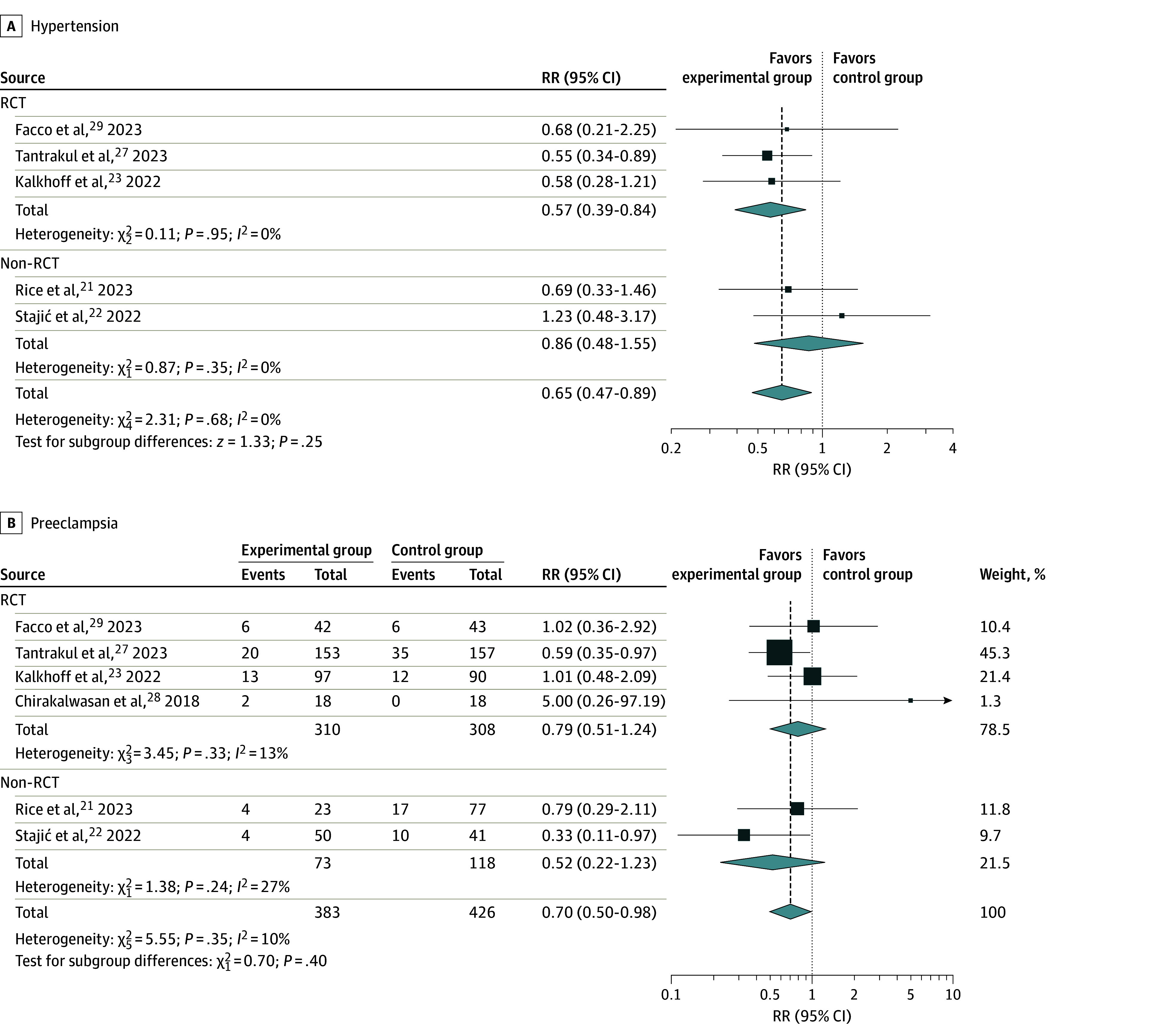
Subgroup Analysis of Type of Study The 95% CI was calculated using random-effects model meta-analysis.

Subgroup analysis was subsequently performed in consideration of the heterogeneity in the CPAP used for treatment. We divided the included trials into 2 subgroups based on classification of the studies: RCTs^23,27,29^ and non-RCTs.^21,22^ The findings revealed a significant reduction in the risk of hypertension in RCTs (RR, 0.57; 95% CI, 0.39-0.84), whereas a similar but not statistically significant outcome was observed in non-RCTs (Figure 3A). Moreover, while there was some reduction in the risk of preeclampsia in both RCTs and non-RCTs, this reduction was not statistically significant (Figure 3B). Meta-regression was performed to examine whether patients’ age and BMI would modify the association with CPAP usage. Patients’ age (coefficient, −0.0190; *P* = .83), and BMI (coefficient, −0.0042; *P* = .87) were not correlated with a reduction of hypertension and preeclampsia risk (eFigure 1 in Supplement 1).

**Figure 4.**
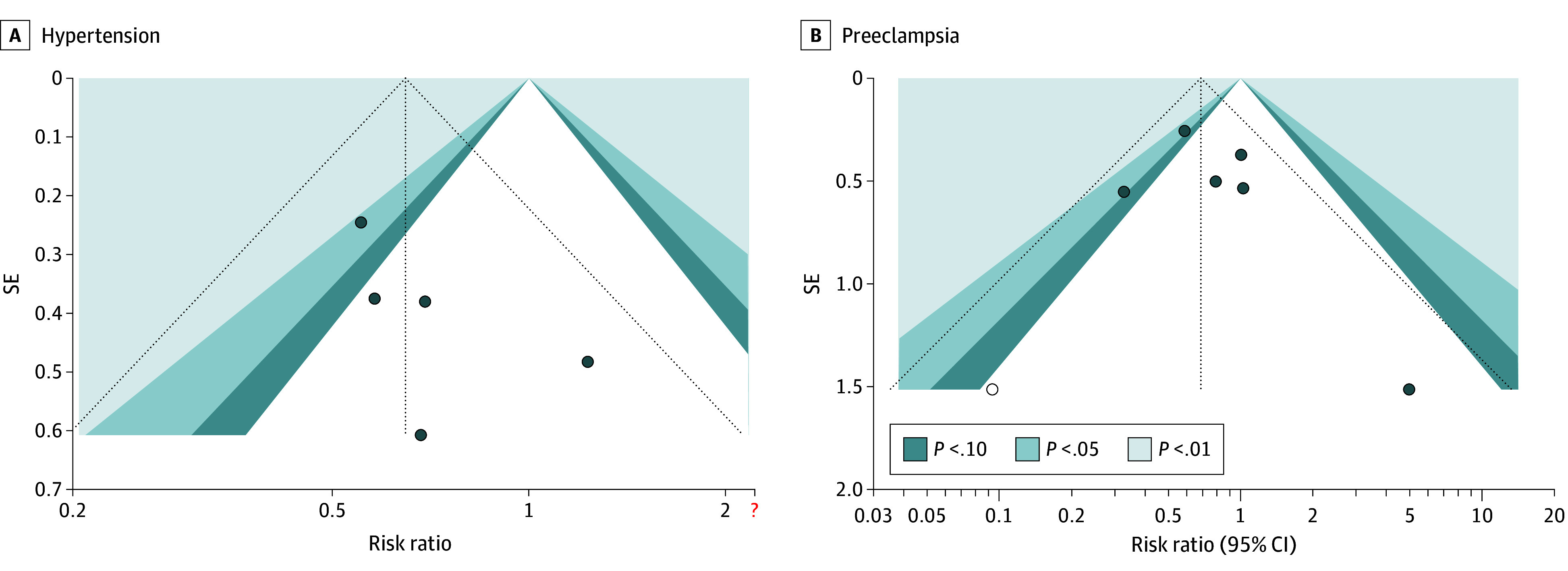
Trim-and-Fill Funnel Plots of Included Trials The black circles represent the observed studies; the white circle in panel B represents the imputed study by the trim-and-fill method. The vertical line represents the overall estimated effect if corrected by the trim-and-fill analysis for publication bias.

The trim-and-fill test was conducted to examine publication bias. Although a skewed funnel plot was seen, the results were unchanged after applying the trim-and-fill method (Figure 4). A sensitivity analysis was conducted using the leave-one-out method. The results consistently showed a statistically significant association with a reduced risk of hypertension except when the study by Tantrakul et al^27^ was omitted. However, the direction of the association remained unchanged. Additionally, the sensitivity analysis for the association of CPAP use with preeclampsia was performed. The association with preeclampsia weakened when the studies by Stajić et al,^22^ Tantrakul et al,^27^ and Rice et al^21^ were individually omitted. Similarly, the direction of association remained unchanged (eFigure 2 in Supplement 1).

## Discussion

This study is a systematic review of existing evidence regarding CPAP treatment for pregnant women with OSA. The meta-analysis focused on evaluating the association of CPAP treatment and the incidence of composite hypertensive outcomes in pregnant women.

The selected studies included 2 observational studies^21,22^ and 4 RCTs,^23,27,28,29^ each with some notable differences. First, there was variability in the study population. Kalkhoff et al^23^ explored the effect of sleep study screening with CPAP treatment in pregnant women, enrolling pregnant women with and without OSA. In contrast, the other 5 studies exclusively enrolled pregnant women with confirmed OSA (apnea-hypopnea index >5 or respiratory event index >5, both indicating at least mild OSA) by either polysomnography or home sleep test.^21,22,27,28,29^ Second, the criteria for CPAP treatment also varied, with 3 studies offering CPAP for women diagnosed with OSA,^27,28,29^ 2 for patients with moderate or mild OSA,^21,23^ and 1 for patients with mild OSA.^22^ Additionally, the timing and duration of CPAP treatment were not clearly stated in some studies. Generally, CPAP treatment was initiated in the second trimester to early third trimester. There was also a range of CPAP treatment compliance, with Kalkhoff et al reporting poor adherence (<10%), while Rice et al^21^ documented 84% adherence. Logically, the true clinical association may be larger with longer adherence to CPAP during pregnancy.

In sensitivity analysis using the leave-one-out method, we observed that the study by Tantrakul et al^27^ had a substantial influence on the pooled outcomes of reducing both hypertension and preeclampsia. This influence may be attributed to its large-scale design, which comprised the largest sample size (311 participants) among the included studies, which led to its carrying the most weight in the pooled analysis. Of note, however, the direction of the summary effect remained unchanged when omitting this study. Additionally, for the reduction in the risk of preeclampsia, the studies by Stajić et al^22^ and Rice et al^21^ also had an influence on the effect size. The evidence supporting the association between CPAP treatment and the reduction in the risk of preeclampsia appears to be inconclusive based on the current studies.

The meta-analysis showed that CPAP treatment in pregnant women with OSA was associated with a significant reduction in the risk of gestational hypertension by 35%. This association may be attributable to the mitigation of sympathetic activity resulting from fragmented sleep by CPAP ventilation, which helps maintain airway patency, thereby reducing frequent arousals and preserving the integrity of sleep architecture.

Preeclampsia is another serious gestational complication. The meta-analysis revealed that CPAP treatment in pregnant women with OSA achieved a significant reduction in the risk of preeclampsia by 30%. This pathway may be largely attributed to the improvement, via CPAP treatment, of endothelial dysfunction from repeated hypoxia. Hypoperfusion of the placenta induces production of vasoconstrictive agents often found in women who develop preeclampsia.^30^ A study showcased that CPAP therapy effectively managed OSA and reduced soluble fms-like tyrosine kinase 1 antiangiogenic factor concentrations in a high-risk pregnancy with chronic hypertension.^31^ This finding supports the connection between placental hypoxia and endothelial dysfunction, which could escalate to preeclampsia. Treatment with CPAP may have positive outcomes for placental physiology.

Indications for CPAP treatment in pregnancy vary, ranging from confirmed OSA diagnosed through polysomnography to the presence of risk factors for preeclampsia, as well as cases of severe preeclampsia requiring hospital admission. Clinically, CPAP treatment during pregnancy is recommended once the diagnosis of OSA is confirmed.^32^ Implementation typically involves shared decision making with full informed consent. However, health care professionals may prioritize CPAP treatment for pregnant women with moderate to severe OSA with desaturation or with OSA comorbid with high-risk pregnancy, such as chronic hypertension, obesity, history of preeclampsia, or gestational diabetes in a previous pregnancy, because they are associated with greater risk of complications for both the mother and fetus during pregnancy.

The timing of CPAP treatment for pregnant women with OSA may vary based on individual circumstances, which are influenced by the severity of OSA and the presence of gestational complications. It is generally recommended that patients with a preexisting OSA diagnosis and established CPAP treatment should continue using CPAP during their pregnancies.^33^ Patients with newly diagnosed OSA should promptly initiate CPAP treatment, typically starting with autotitrating CPAP in most cases.^33^ The application of a CPAP intervention varies widely, ranging from nightly CPAP in early pregnancy to a 4-week block of CPAP between 24 and 28 weeks or CPAP initiation from the diagnosis of gestational diabetes in the third trimester.^32^

Selecting the appropriate type of CPAP mask for pregnant women with OSA is crucial for improving compliance and adherence to therapy. Currently, there is limited robust evidence identifying the most suitable mask for CPAP therapy during pregnancy. Our clinical experience with Asian pregnant women suggests that a pillow cushion with a frame and headgear positioned over the head may offer greater comfort during pregnancy. This configuration allows for increased freedom of movement, especially when changing sleeping positions, such as transitioning to a lateral sleep posture. Nonetheless, it is essential for pregnant women to collaborate closely with their sleep specialists to identify an optimal mask that fits well, facilitates effective therapy, and ensures comfort throughout the treatment process.

### Limitations

This meta-analysis study has some limitations. First, the number of eligible articles was small, limiting reliability and clinical generalizability. Second, the duration of and adherence to CPAP treatment were not clearly stated in all the studies. Given that adherence is a crucial issue in CPAP treatment, variations in adherence among studies could make it challenging to draw a strong conclusion about the treatment’s benefits. Based on current knowledge about OSA in pregnancy and its associated adverse outcomes, screening women with high-risk pregnancies for OSA, followed by CPAP treatment, may reduce the incidence of composite hypertensive syndromes without apparent safety issues.

## Conclusions

Pregnant women may develop or experience an exacerbation of OSA, which may increase the risk of gestational complications. This meta-analysis found that CPAP treatment for pregnant women with OSA was significantly associated with a reduction in the risk of gestational hypertension and preeclampsia. Future research should prioritize assessing treatment adherence and exploring the optimal timing and duration of CPAP use.

## Notes

Supplement 1. **eTable.** Detailed Quality Assessment of Included Studies Using Cochrane Risk of Bias 2 Tool and Newcastle-Ottawa Score **eFigure 1.** Meta-Regression Bubble Plots of the Correlation Between Effect of Risk Reduction and Age and Body Mass Index **eFigure 2.** The Results of a Sensitivity Analysis Using the One-Study Removal Method

Supplement 2.
Data Sharing Statement
