# Supplementary material for: Continuous Positive Airway Pressure Treatment and Hypertensive Adverse Outcomes in Pregnancy: A Systematic Review and Meta-Analysis
Source: JAMA Netw Open. 2024 Aug 13;7(8):e2427557. doi: 10.1001/jamanetworkopen.2024.27557 (PMC11322849; doi:10.1001/jamanetworkopen.2024.27557)
Supplement: Supplement 1. — eTable. Detailed Quality Assessment of Included Studies Using Cochrane Risk of Bias 2 Tool and Newcastle-Ottawa Score eFigure 1. Meta-Regression Bubble Plots of the Correlation Between Effect of Risk Reduction and Age and Body Mass Index eFigure 2. The Results of a Sensitivity Analysis Using the One-Study Removal Method [file jamanetwopen-e2427557-s001.pdf]

# Supplemental Online Content

Lee Y-C, Chang Y-C, Tseng L-W, et al. Continuous positive airway pressure treatment and hypertensive adverse outcomes in pregnancy: a systematic review and meta-analysis. *JAMA Netw Open*. 2024;7(8):e2427557. doi:10.1001/jamanetworkopen,2024.27557

**eTable.** Detailed Quality Assessment of Included Studies Using Cochrane Risk of Bias 2 Tool and Newcastle-Ottawa Score

**eFigure 1.** Meta-Regression Bubble Plots of the Correlation Between Effect of Risk Reduction and Age and Body Mass Index

**eFigure 2.** The Results of a Sensitivity Analysis Using the One-Study Removal Method

This supplemental material has been provided by the authors to give readers additional information about their work.

© 2024 Lee Y-C et al. *JAMA Network Open.*

**eTable.** Detailed Quality Assessment of Included Studies Using Cochrane Risk of Bias 2 Tool and Newcastle-Ottawa Score

| First Author | Year | Randomization process | Intervention adherence | Missing outcome data | Outcome measurement | Selective reporting | Overall RoB |
|---|---|---|---|---|---|---|---|
| Tantrakul et al.[27] | 2023 | L | L | L | L | L | L |
| Facco et al.[29] | 2023 | L | L | L | L | L | L |
| Kalkhoff et al.[23] | 2022 | L | L | L | L | L | L |
| Chirakalwasan et al.[28] | 2018 | L | L | L | L | L | L |

| First Author | Year | Selection | Comparability | Outcome | Newcastle–Ottawa total score |
|---|---|---|---|---|---|
| Rice et al.[21] | 2022 | 4 | 0 | 2 | 6 |
| Stajic et al.[22] | 2021 | 4 | 0 | 2 | 6 |

© 2024 Lee Y-C et al. *JAMA Network Open.*

**eFigure 1.** Meta-Regression Bubble Plots of the Correlation Between Effect of Risk Reduction and (a)Age (b)Body Mass Index. Each bubble represents a study and bubble size represents the sample size of the study.((a) age, coefficient = -0.0190, *p* =0.83. (b) BMI, coefficient = -0.0042, *p* = 0.87.)

(a) Age

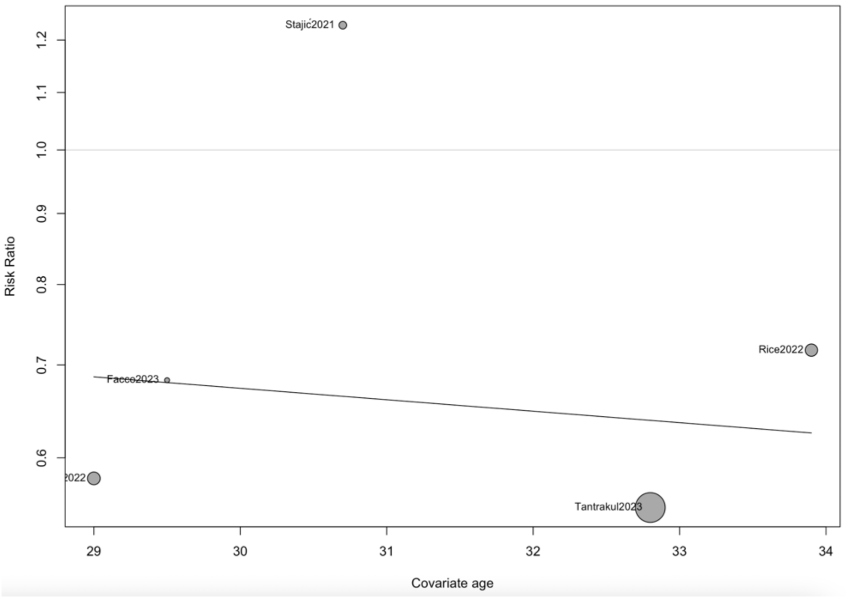


(b) BMI

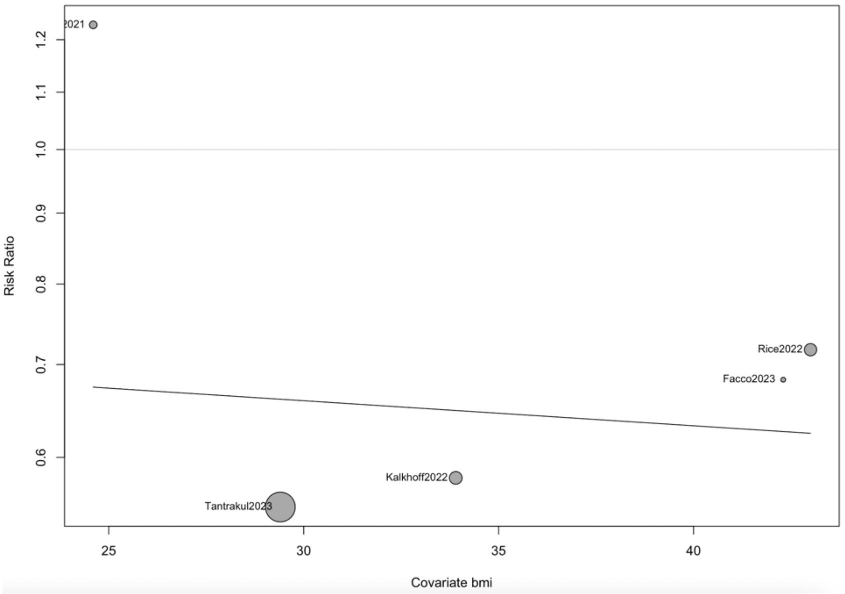


© 2024 Lee Y-C et al. *JAMA Network Open.*

**eFigure 2.** The Results of a Sensitivity Analysis Using the One-Study Removal Method. The main result did not change significantly after removing any one of the included trials, showing effects of continuous positive pressure treatment on (a) hypertension (b) pre-eclampsia. CI, confidence interval.

(a) hypertension

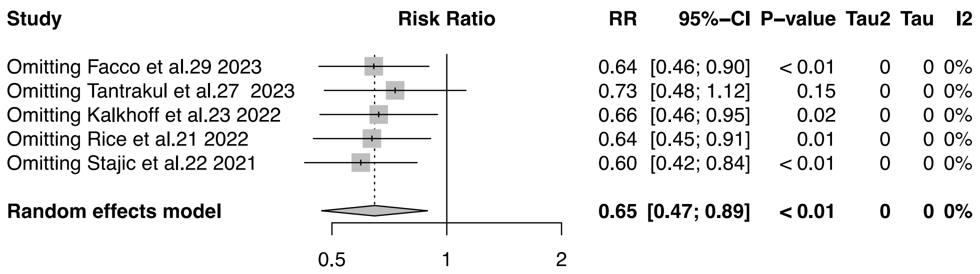


(b) pre-eclampsia

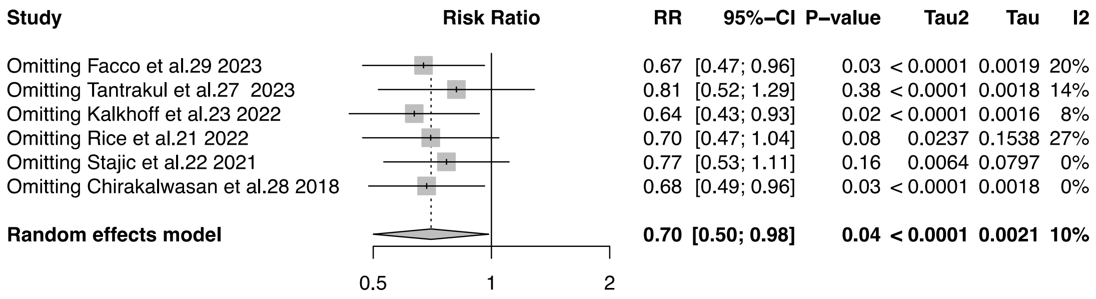


© 2024 Lee Y-C et al. *JAMA Network Open.*
