# Supplementary material for: Continuous Positive Airway Pressure Treatment and Hypertensive Adverse Outcomes in Pregnancy: A Systematic Review and Meta-Analysis
Source: JAMA Netw Open. 2024 Aug 13;7(8):e2427557. doi: 10.1001/jamanetworkopen.2024.27557 (PMC11322849; doi:10.1001/jamanetworkopen.2024.27557)
Supplement: Supplement 2. — Data Sharing Statement [file jamanetwopen-e2427557-s002.pdf]

Data Sharing Statement

Lee. Continuous Positive Airway Pressure Treatment and Hypertensive Adverse Outcomes in Pregnancy. *JAMA Netw Open*. Published August 13, 2024.
doi:10.1001/jamanetworkopen.2024.27557

Data
Data available: No
